# Associations of aortic and carotid artery health with cerebrovascular markers and cognition in older adults from the Whitehall II imaging study

**DOI:** 10.1186/s12916-025-04105-y

**Published:** 2025-06-03

**Authors:** Georgina Hobden, Graham Reid, Scott T. Chiesa, Congxiyu Wang, Lucy Jobbins, Clare E. Mackay, Alun D. Hughes, Klaus P. Ebmeier, Jemma Pitt, Mika Kivimäki, Archana Singh-Manoux, Sana Suri

**Affiliations:** 1 Department of Experimental Psychology, University of Oxford, Oxford, UK; 2 Department of Psychiatry, University of Oxford, Oxford, United Kingdom; 3 Department of Population Science and Experimental Medicine, Institute of Cardiovascular Science, UCL, London, UK; 4 Oxford Centre for Human Brain Activity, Wellcome Centre for Integrative Neuroimaging, University of Oxford, Oxford, UK; 5 UCL Brain Sciences, University College London, London, UK; 6 Epidemiology of Ageing and Neurodegenerative Diseases, Université Paris Cité, INSERM U1153 Paris, France

**Keywords:** Dementia, Carotid, Aorta, Cognition, Ultrasound, Magnetic resonance imaging, Cardiovascular risk factors, Longitudinal cohort

## Abstract

**Background:**

Cardiovascular disease has been associated with an increased dementia risk, but the underlying mechanisms for this heart-brain link are unclear. This study sought to examine associations between aortic and carotid artery structure with cerebrovascular reactivity (CVR), white matter hyperintensities (WMHs), and cognition in later-life.

**Methods:**

One hundred sixty three participants (25.8% female) from the Whitehall II Imaging cohort completed two examinations (*M* ± *SD* age 68.2 ± 4.4 at Wave-1 and 76.9 ± 4.5 at Wave-2) of neuropsychological assessments and 3T brain magnetic resonance imaging (MRI) FLAIR scans to quantify WMHs. Wave-2 additionally included vascular sonography of the aorta and carotid artery, and 3T functional MRI scans to measure CVR (mean % change BOLD signal change during a CO_2_ challenge). Wave-2 factor scores of aortic and carotid arterial diameters, stiffness, and compliance were the exposure variables. Midlife Framingham Cardiovascular Risk Score (FRS) measured before Wave-1 was a potential effect modifier. WMH volume, grey matter CVR, cognitive factor scores (episodic memory, working memory, executive function, visuospatial memory, fluency, lexical retrieval) at Wave-2, and changes in WMH and cognition between Wave-1 and Wave-2 were used as outcome variables.

**Results:**

Larger aortic diameter (ß = 0.38, *SE* = 0.11) and greater aortic stiffness (ß = 0.27, *SE* = 0.10) were associated with larger carotid diameter, independently of body size. Higher midlife FRS was associated with larger aortic and carotid diameters and increased carotid stiffness in old age. We observed notable artery-brain associations, such that larger aortic (ß = 0.17, *SE* = 0.06) and carotid diameters (ß = 0.11, *SE* = 0.05) were associated with larger WMH lesion volumes at Wave-2. Larger aortic diameter (ß = 0.08 *SE* = 0.03) and lower carotid compliance (ß = − 0.06, *SE* = 0.02) at Wave-2 were also associated with greater longitudinal increases in WMH volumes over the preceding 9 years. Higher stiffness and lower compliance of the aorta and carotid were associated with worse cognitive outcomes across a range of domains, and these associations were moderated by midlife FRS. Larger carotid diameter was associated with higher cerebrovascular reactivity (ß = 0.02, *SE* = 0.01), suggesting a potential compensatory pathway.

**Conclusions:**

Adverse structural and functional changes in the aorta and carotid artery were inter-related and associated with vascular brain lesions, cerebrovascular reactivity, and poorer cognitive outcomes in older age.

**Supplementary Information:**

The online version contains supplementary material available at 10.1186/s12916-025-04105-y.

## Background

Dementia and cardiovascular disease (CVD) pose significant healthcare challenges. Given the shared vascular risk factors for both conditions, and the evidence pinpointing midlife as a critical period to reduce cardiovascular risk for dementia [1], exploring heart-brain links during mid-to-old age has become an important area of research [2]. The heart and brain are connected via complex arterial pathways, with the brain receiving part of its blood supply from the common carotid and vertebral arteries in the neck, which are in turn supplied by the aorta leaving the heart [3]. Existing research has focused on aorta-brain links, revealing associations between lower aortic distensibility (elasticity) and larger volumes of cerebral white matter hyperintensities (WMHs) [4–6], with the latter increasing risk of cognitive impairment and dementia [7]. Lower aortic distensibility has also been associated with greater longitudinal increases in WMHs [8, 9] and faster conversion from mild cognitive impairment to dementia [10–12]. Aortic stiffening, in turn, has been linked to poorer cerebral blood flow and cognition [13], as well as faster long-term decline in cognitive functioning [14]. The mechanistic underpinnings of these associations are unclear, but it is likely that loss of vessel elasticity and a resultant increase in pulse wave velocity across the cardiac cycle can contribute to higher wave pressure during perfusion of the brain. This can damage the delicate cerebral microvasculature and contribute to the buildup of vascular lesions (white matter hyperintensities) and neuronal damage. Cerebral autoregulatory mechanisms, such as cerebrovascular reactivity (CVR), could help protect against pressure variability from the heart by dilating or contracting the brain’s blood vessels to meet metabolic demands [15]. However, while impairments in CVR are known to contribute to cerebral damage, cognitive decline, and dementia [16–21], we know little about how CVR is affected by age-related changes in aorta and large artery physiology.

Moreover, very few studies have investigated associations between the carotid artery and later-life brain and cognitive outcomes [22, 23], despite these arteries directly supplying the brain [3]. For example, carotid plaques have been linked with higher WMHs and poor memory performance [24, 25], and carotid wave intensity with cognitive decline [22]. But almost no neuroimaging studies have examined the aorta and carotid arteries together. It is possible that any carotid-brain associations may derive from damage to the aorta, but it may also be that the transmission of pulsatile load from the carotids to the brain is independent of aortic stiffness, given evidence suggesting that aortic and carotid artery distensibility may not necessarily correlate [26, 27]. Examining aortic and carotid phenotypes together would therefore enable better understanding of how peripheral circulation relates to cerebrovascular and cognitive function.

This study examined the association between aortic and carotid artery structure with brain and cognitive outcomes, using data from the Heart and Brain Study on the Whitehall II cohort participants [28]. We conducted comprehensive ultrasound imaging, brain magnetic resonance imaging (MRI), and cognitive assessments at two waves. At MRI-Wave-1 (age 68.2 ± 4.4 years), we measured WMHs and cognitive performance. At MRI-Wave-2 (age 76.9 ± 4.5 years), we acquired repeat measures of WMH and cognition, as well as CVR scans and three summary measures for the aortic and carotid arteries: distensibility (expansion ability with increasing transmural pressure), stiffness (rigidity), and diameter (distance between opposing vessel walls). We examined cross-sectional associations of artery measures at Wave-2 with (1) cerebrovascular reactivity (CVR), WMH volume, and cognition at Wave-2 and (2) changes in WMHs and cognitive function during the preceding 9 years (between Waves 1 and 2). Our objective was to determine whether arterial measures were associated with 1) better concurrent brain and cognitive function and (2) better preservation of brain function during the past decade. We hypothesised that structural decline of the major arteries connecting the heart and brain would be linked to poorer neuro-cognitive outcomes. Given the growing emphasis on early prevention and vascular risk reduction in midlife, we also measured the Framingham Cardiovascular Disease Risk Score (FRS, which combines CVD and dementia risk factors) at 40–60 years old [29, 30] and examined whether overall cardiovascular risk in midlife, as assessed with this score, may modify later-life artery-brain associations.

## Methods

### Participants

This study used data from the Heart and Brain Study [28], and involved 163 participants from the Whitehall II cohort [31]. Data were collected at the University of Oxford’s Wellcome Centre for Integrative Neuroimaging (MRI-Wave-1 at the Centre for Functional Magnetic Resonance Imaging of the Brain (2012–2016, MSD/IDREC/2010/P17.2; detailed study protocol: [32]) and MRI-Wave-2 at the Oxford Centre for Human Brain Activity (2019–2023; R57135/RE006; detailed study protocol: [28]). MRI-Wave-1 included cognitive assessments and 3T MRI scans to measure WMHs. MRI-Wave-2 included repeated cognitive and WMHs measures from Wave 1, as well as additional vascular ultrasound scans (aorta and carotid), and an MRI scan to measure cerebrovascular reactivity (CVR). CVR and vascular ultrasound scans were acquired at Wave-2, but not at Wave-1. The Framingham risk score was assessed at Whitehall II UCL clinic in 1991–1994 and 1997–1999. In the current analyses, MRI-Wave-I is used as baseline and MRI-Wave-2 is used as follow-up.

Participants for MRI-Wave-2 were included in the analyses if they participated in the 2019 wave of the Whitehall II Study, had good-quality scans without significant incidental findings at MRI-Wave-1, had no MRI contraindications, no contraindications to the hypercapnia challenge, and no diagnosis of dementia (Additional file 1: Figure S1).

### Vascular sonography

Aortic scans (Additional file 1: Method S1) of the proximal ascending aorta used a 4-MHz cardiac transducer on a GE VIVID 7 system (November 2019–January 2023, *n* = 112). Carotid scans (Additional file 1: Method S2) used a 13-MHz linear transducer on a GE VIVID 7 system (November 2019–January 2023, *n* = 112) and Zonare Z.One System (January 2023–May 2023, *n* = 51). Aortic and left and right carotid measures included arterial diameter (mm), pulse wave velocity (m/s), arterial compliance (mm^2^/kPA), distensibility (10^−3^ kPa), and beta stiffness index (equations in Additional file 1: Method S3). All participants underwent scanning of the left and right common carotid arteries, and due to a scanner change partway through the study, only a subset of participants underwent additional scanning of the ascending aortic artery. Left and right carotid measures were averaged to provide a single overall measure for the carotids. Summary measures were derived using principal component analysis (PCA, see details in Additional file 1: Figure S2).

### Brain MRI

MRI-Wave-1 scans (3T Siemens Magnetom Verio, Apr 2012–Dec 2015, 32-channel head coil) and MRI-Wave-2 scans (3T Siemens Prisma, Nov 2019–May 2023, 64-channel head coil) had closely matched protocols. This study used CVR scans from MRI-Wave-2 and fluid attenuated inversion recovery (FLAIR) scans from both MRI-Waves-1 and 2. T1 MPRAGE scans (1 mm3, TR = 1900 ms, TE = 3.97 ms, TI = 904 ms, flip angle = 8°) from both waves were used for registration and image processing. Data were pre-processed using FMRIB Software Library (FSL) tools [33] and visually inspected using FSLeyes [34]. For details of the brain measures, see Additional file 1: Method S4.

#### Cerebrovascular reactivity (CVR)

CVR, measured as the blood-oxygen-level-dependent (BOLD) response to 5% carbon dioxide (CO_2_), involved participants wearing a mask supplying normocapnic air (0.04% CO_2_, 60 s), followed by two 75-s blocks of hypercapnic air (5% CO_2_), interleaved with two 75-s blocks of normocapnic air (0.04% CO_2_) [28]. This block design has been extensively piloted and validated and has been described in detail in the study protocol paper [28]. The mask was connected to a PowerLab 4/35 data system and ML206 gas analyser to measure inspired and expired gas concentrations (ADInstruments, New Zealand). BOLD gradient-echo echoplanar imaging sequences were acquired (2.4 mm3 resolution, TR = 800 ms, TE = 30 ms, 450 volumes, flip angle = 52°). Processing included motion correction, spatial smoothing (4 mm kernel), and high-pass temporal filtering with FSL-FEAT (210 Hz) [33]. A MATLAB script extracted the end-tidal CO_2_ (EtCO_2_; exhaled CO_2_ concentrations) from participants’ CO_2_ traces, yielding a normalised value per fMRI volume that was aligned to the BOLD time-course. For each participant, mean %BOLD signal change was extracted from the whole-brain grey matter as well as the frontal, temporal, parietal, and occipital lobe grey matter using Featquery. CVR (% BOLD per mmHg) was calculated by dividing the mean %BOLD signal change by the change in EtCO_2_ (average maximum EtCO_2_ across two hypercapnic blocks minus average baseline EtCO_2_).

#### FLAIR

T2-weighted FLAIR scans (0.4 × 0.4 × 3 mm, TR = 9000 ms, TE = 73 ms, flip angle = 150°) at both waves underwent automated WMH segmentation and quantification using FSL-BIANCA [35]. WM masks in T1 space were registered to FLAIR space, and FSL-BIANCA was run on masked FLAIR and brain-extracted T1 images, and a FLAIR-to-MNI matrix as inputs. Probability maps were thresholded at 0.9 to extract WMH volumes (mm^3^). Volumes were normalized as a percentage of total brain volume and log-transformed because of skewness (logWMH) [35]. Longitudinal change in WMH volumes between the two timepoints was calculated as the difference in logWMH between waves (ΔlogWMH). We also repeated our analyses without log transforming the WMH values and observed similar results. Given previous research on this cohort has used log-transformed WMH%, we present results below using transformed data only [35].

### Cognition

We administered the same battery of cognitive tests at both study Waves: Trail Making Test Versions A and B (assessing speed of processing and executive function) [36], Hopkins Verbal Learning Test-Revised (assessing verbal episodic memory) [37], Digit Span Test (assessing short-term memory) [38], Digit Coding Test (assessing short-term memory and executive function) [38], Verbal Fluency Test [39], Rey Complex Figure Test (used to assess visuospatial memory, planning, attention) [40], Boston Naming Test (used to assess semantic retrieval) [41], and the Test of Premorbid Functioning scaled to full scale IQ (assessing premorbid intellectual ability and lexical retrieval) [42]. These tests are part of standard neuropsychological batteries, have been extensively validated, and show sensitivity for cognitive changes in this age range. Details of the tests are presented in the protocol papers for MRI-Wave-1 [32] and MRI-Wave-2 [28], as well as in Additional file 1: Method S5. We performed a principal component analysis (PCA) to combine large quantities of data from the cognitive tests and subscales into meaningful cognitive domains and reduce the dimensionality (detailed derivations are described below and in Additional file 1: Figure S2).

### Framingham risk score

Midlife FRS was calculated at age 40–65 (from the 1991–1994 wave, or, 1997–1999 wave of the Whitehall II Study) [43]. This estimates cardiovascular disease risk based on age, sex, smoking, cholesterol (total and high-density lipoprotein; HDL), systolic blood pressure, and anti-hypertensive treatment. Validated in the Whitehall II study [44], FRS has been shown to predict both cognitive decline [45] and dementia progression [46], and in these analyses, participants were dichotomized as low risk (FRS < 10) or moderate-high risk (FRS ≥ 10) [44]. Moderate and high risk were combined as only 3 participants were in the high risk group (i.e., scoring FRS > 20).

### Statistical analysis

Analyses were performed in RStudio. Outliers were removed based on values > 6 * median absolute deviation. PCAs with *oblimin* rotation (i.e., an oblique rotation method that permits factors to be intercorrelated) were conducted using *psych v2.3.6* [47] to derive factors for aortic and carotid measures and cognitive tests. Five artery measures were entered into separate PCAs for the aortic and carotid arteries and sixteen cognitive measures were entered into a PCA for cognition. Based on factor loadings, scree plots, eigenvalues, and cumulative explained variance, we derived three factors each for the carotid and aorta: stiffness (comprising beta stiffness index and pulse wave velocity), diameter (systolic and diastolic diameter), compliance (comprising distensibility and compliance) (Additional file 1: Figure S2).

We derived six cognitive factors: episodic memory, working memory, executive function, visuospatial memory, verbal fluency, and lexical retrieval (Additional file 1: Figure S2). Factor weights from MRI-Wave-2 were applied to the MRI-Wave-1 cognitive scores, assuming factor loadings are time-independent [48]. Longitudinal change in each factor (Δ of the six cognitive domains) was measured as the difference between the respective factors derived at MRI-Wave-1 and MRI-Wave-2. For executive function and visuospatial memory, higher factor values represent poorer performance, whereas for episodic memory, working memory, and lexical retrieval, lower values represent poorer performance.

We used ANCOVAs to compare aortic and carotid factors between FRS groups, correcting for age, sex, and height. Longitudinal changes in WMH volume and cognition were assessed using paired *t*-tests. We used linear regression to examine associations between exposures (aortic or carotid factors) and outcomes (logWMH at MRI-Wave-2, ΔlogWMH (Wave-2 – Wave-1), CVR at MRI-Wave-2, cognitive factors at MRI-Wave-2, and Δcognition (Wave-2 – Wave-1). Based on the number of exposures and outcomes, multiple comparisons were corrected using the Benjamini-Hochberg (BH) correction, defining significance as *p*_corr_ < 0.05. We also present significant results (*p* < 0.05) that did not survive this correction.

All models included sex, years of education, age, and body size (height in m) at MRI-Wave-2 as covariates. Arterial phenotypes such as diameter are known to be associated with body size [49], and so we used height to control for taller people having larger arteries. We also repeated the analysis correcting for BMI instead of height and this made no difference to our results. Longitudinal analyses also included time between waves as a covariate. Carotid analyses additionally included ultrasound scanner model as a covariate. Midlife FRS (moderate versus low risk) was used as a moderator to stratify the regression analyses.

## Results

### Participant characteristics

Among the 163 participants, 42 (25.8%) were women, and the mean (SD) age was 68.2 (4.4) years at MRI-Wave-1 and 76.9 (4.5) years at MRI-Wave-2 (Additional file 1: Figure S1 and demographics in Table 1). The mean (SD) time between MRI-Wave-1 and MRI-Wave-2 testing was 8.7 (1.3) years. Aorta scans were available for 89 participants due to change in equipment during the study, and carotid scans for 153 participants (Additional file 1: Figure S1 and demographics in Table 1). For a summary of demographic variables across the low FRS and moderate-high FRS risk groups, see Additional file 1: Table S1.

**Table 1:** Demographic characteristics and exposures for the overall study sample (*n* = 163) and subsamples included in the aortic (*n* = 89) and carotid (*n* = 153) analyses

Variable	Overall sample *N* = 163	Aorta subsample *N* = 89	Carotid subsample *N* = 153
Gender, *N* (%)			
*Female*	42 (25.8)	21 (23.6)	41 (26.8)
*Male*	121 (74.2)	68 (76.4)	112 (73.2)
Age at MRI-Wave-2 (years), *M* (*SD*)	76.9 (4.5)	76.7 (4.7)	76.7 (4.5)
Education (years), *M* (*SD*)	15.0 (3.6)	14.7 (3.5)	15.08 (3.6)
Time between MRI-Wave-1 and MRI-Wave-2 (years), *M* (*SD*)	8.67 (1.27)	8.26 (1.17)	8.69 (1.24)
BMI at MRI-Wave-2 (kg/m^2^), *M* (*SD*)	25.9 (4.3)	26.0 (4.2)	25.7 (4.3)
FRS (1991–1994, or 1997–1999), *N* (%)			
*Low*	124 (76.1)	65 (73)	118 (77.1)
*Moderate-high*	38 (23.3)	24 (27)	34 (22.2)
Systolic BP (mmHg), *M* (*SD*)	149.6 (19.2)	149.8 (19.9)	149.5 (18.9)
Diastolic BP (mmHg), *M* (*SD*)	78.34 (10.9)	78.66 (11.7)	78.42 (10.9)
Pulse pressure, *M* (*SD*)	71.29 (16.0)	71.09 (15.8)	71.05 (15.6)
Ultrasound scanner, *N* (%)			
*GE VIVID 7*	111 (68.1)	89 (100)	103 (67.3)
*ZONARE Z.One*	52 (31.9)	0 (0)	50 (32.7)
Aortic diameter (mm), *M* (*SD*)	34.1 (3.7)	34.1 (3.7)	34.1 (3.7)
Aortic pulse wave velocity (m/s), *M* (*SD*)	12.8 (3.6)	12.9 (3.5)	12.92 (3.6)
Aortic compliance (mm^2^/kPA), *M* (*SD*)	6.4 (3.8)	6.4 (3.8)	6.17 (3.7)
Aortic distensibility coefficient, *M* (*SD*)	7.1 (3.9)	7.1 (3.9)	7.0 (3.9)
Aortic beta stiffness index, *M* (*SD*)	25.3 (14.3)	25.6 (14.2)	25.8 (14.2)
Carotid diameter (mm), *M* (*SD*)	8.0 (0.9)	8.0 (0.9)	8.0 (0.9)
Carotid pulse wave velocity (m/s), *M* (*SD*)	9.6 (1.5)	9.7 (1.6)	9.6 (1.5)
Carotid compliance (mm^2^/kPA), *M* (*SD*)	0.6 (0.2)	0.6 (0.2)	0.6 (0.2)
Carotid distensibility coefficient, *M* (*SD*)	11.4 (3.5)	11.1 (3.5)	11.4 (3.5)
Carotid beta stiffness index, *M* (*SD*)	13.7 (4.2)	14.0 (4.5)	13.7 (4.2)

Values were obtained at MRI-Wave-2 unless otherwise specified

*T*-tests showed between-wave increases in WMH volume (*t* = − 19.07, Cohen’s *d* = 1.5, *p* < 0.001) and decreases in episodic memory (*t* = − 27.17, *p* < 0.001, Cohen’s *d* = − 2.1), visuospatial memory (*t* = 4.48, *p* < 0.001, Cohen’s *d* = 0.36), fluency (*t* = − 2.15, *p* = 0.03, Cohen’s *d* = − 0.17), executive function (*t* = 15.00, *p* < 0.001, Cohen’s *d* = 1.2), working memory (*t* = − 13.32, *p* < 0.001, Cohen’s *d* = − 1.0), and lexical retrieval (*t* = − 13.26, *p* < 0.001, Cohen’s *d* = − 1.0; Additional file 1: Table S2). Decreases in performance are represented as increased scores for visuospatial memory and executive function memory from Wave 1 to 2 but decreased scores for the other cognitive domains.

Larger aorta diameter (ß = 0.38, 95% CI [0.17 0.61], *p* < 0.001, *p*_corr_ < 0.05) and higher aortic stiffness (ß = 0.27, 95% CI [0.08 0.47], *p* = 0.006, *p*_corr_ < 0.05) were associated with larger carotid diameter (Additional file 1: Figure S3).

**Fig. 1:**
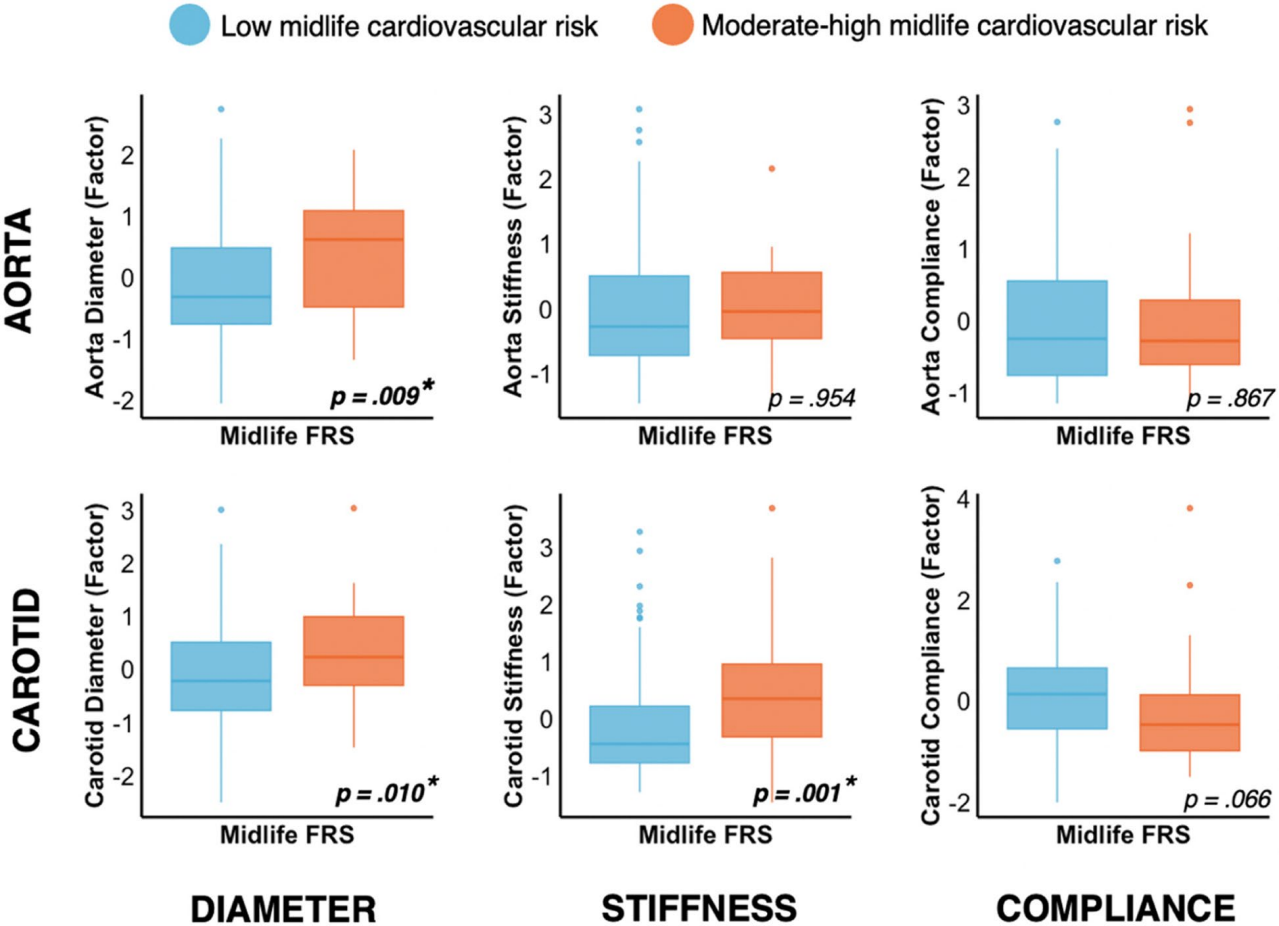
Association of aorta and carotid phenotypes with midlife Framingham risk (blue: low risk group, orange: moderate-high risk). *indicates *p*_corr_ < 0.05, corrected for multiple comparisons

Participants with higher Framingham scores (FRS) in midlife had larger aortic diameter (η^2^ = 0.03, *F*(1,83) = 7.13, *p* = 0.009), larger carotid diameter (η^2^ = 0.003, *F*(1,146) = 6.77, *p* = 0.010), and higher carotid stiffness (η^2^ = 0.01, *F*(1,146) = 10.44, *p* = 0.001) at MRI-Wave-2 after covarying for age, sex, and height (Fig. 1).

Summary statistics for associations of aorta-carotid measures (Table 2), artery-brain measures (Table 3), and artery-cognitive measures (Table 4) are below. A supplementary analysis to determine the reproducibility of the results with a consistent sample size can be found in Additional file 1: Table S3.

**Table 2:** Summary statistics for associations between aortic and carotid factors at MRI-Wave-2 (n = 86)

		Aorta		
		Compliance	Diameter	Stiffness
**Carotid**	**Compliance**	β = 0.07, SE = 0.11, *p* = 0.54	β = 0.19, SE = 0.13, *p* = 0.13	β = 0.07, SE = 0.11, *p* = 0.54
	**Stiffness**	β = − 0.10, SE = 0.11, *p* = 0.37	β = − 0.08, SE = 0.13, *p* = 0.51	β = 0.05, SE = 0.11, *p* = 0.65
	**Diameter**	β = − 0.07, SE = 0.10, *p* = 0.48	β = 0.38, SE = 0.11, *p* = 0.001*	β = 0.27, SE = 0.10, *p* = 0.01*

Note. Arterial compliance (mm^2^/kPA). Arterial diameter (mm)

All models were adjusted for sex, years of education, age, and height at MRI-Wave-2. *ß* Unstandardised regression coefficients, *SE* Standard errors. ^*^indicates p_corr_ < 0.05, corrected for multiple comparisons. Uncorrected significant associations are emboldened

**Table 3:** Summary statistics for associations between aortic and carotid factors with brain outcomes

	**Aorta**		
	**Compliance**	**Diameter**	**Stiffness**
**WMH**	β = − 0.02, SE = 0.05, *p* = 0.70	β = 0.17, SE = 0.06, *p* = 0.005*	β = 0.01, SE = 0.05, *p* = 0.83
**Δ WMH**	β = − 0.03, SE = 0.03, *p* = 0.31	β = 0.08, SE = 0.03, *p* = 0.008*	β = 0.02, SE = 0.03, *p* = 0.38
**CVR**	β = 0.02, SE = 0.01, *p* = 0.06	β = 0.02, SE = 0.01, *p* = 0.05	β = − 0.02, SE = 0.01, *p* = 0.10
	**Carotid**		
	**Compliance**	**Diameter**	**Stiffness**
**WMH**	β = − 0.06, SE = 0.05, *p* = 0.24	β = 0.11, SE = 0.05, *p* = 0.03	β = 0.06, SE = 0.05, *p* = 0.25
**Δ WMH**	β = − 0.06, SE = 0.02, *p* = 0.02*	β = 0.03, SE = 0.02, *p* = 0.18	β = 0.04, SE = 0.02, *p* = 0.06
**CVR**	β = 0.001, SE = 0.01, *p* = 0.92	β = 0.02, SE = 0.01, *p* = 0.04	β = − 0.001, SE = 0.01, *p* = 0.94

Note. Arterial compliance (mm^2^/kPA). Arterial diameter (mm). WMH volume (mm^3^). CVR (% BOLD per mmHg)

All models were adjusted for sex, years of education, age, and height at MRI-Wave-2. Carotid models were also adjusted for ultrasound scanner type. Models investigating change in WMH volume between MRI-Wave-1 and MRI-Wave-2 were also adjusted for time between the two testing sessions. Measures refer to MRI-Wave-2 unless otherwise stated. *ß* Unstandardised regression coefficients, SE Standard errors p, *p* values prior to multiple comparisons correction. Uncorrected significant associations are present in bold. ^*^indicates p_corr_ < 0.05, corrected for multiple comparisons

**Table 4:** Summary statistics for associations between aortic and carotid factors with cognitive outcomes

	**Aorta**		
	**Compliance**	**Diameter**	**Stiffness**
**Episodic Memory**	β = − 0.58, SE = 0.84, *p* = 0.49	β = − 1.31, SE = 0.99, *p* = 0.18	β = − 0.34, SE = 0.87, *p* = 0.69
**Visuospatial Memory**	β = − 1.01, SE = 1.5, *p* = 0.50	β = − 0.5, SE = 1.75, *p* = 0.78	β = − 0.22, SE = 1.54, *p* = 0.89
**Working Memory**	β = 0.42, SE = 0.54, *p* = 0.44	β = 0.72, SE = 0.62, *p* = 0.25	β = − 0.23, SE = 0.55, *p* = 0.68
**Lexical Retrieval**	β = 1.21, SE = 0.88, *p* = 0.18	β = − 1.4, SE = 1.03, *p* = 0.18	β = − 2.68, SE = 0.86, *p* = 0.003 *
**Verbal Fluency**	β = 1.2, SE = 0.91, *p* = 0.19	β = 0.38, SE = 1.06, *p* = 0.72	β = − 1.23, SE = 0.93, *p* = 0.19
**Executive Function**	β = 0.7, SE = 3.81, *p* = 0.86	β = 2.66, SE = 4.42, *p* = 0.55	β = − 2.3, SE = 3.87, *p* = 0.56
**Δ Episodic Memory**	β = − 0.97, SE = 0.8, *p* = 0.23	β = − 1.27, SE = 0.94, *p* = 0.18	β = 0.19, SE = 0.81, *p* = 0.81
**Δ Visuospatial Memory**	β = − 0.98, SE = 1.45, *p* = 0.50	β = − 2.48, SE = 1.71, *p* = 0.15	β = − 0.29, SE = 1.48, *p* = 0.85
**Δ Working Memory**	β = 0.18, SE = 0.42, *p* = 0.67	β = − 0.12, SE = 0.49, *p* = 0.80	β = − 0.08, SE = 0.42, *p* = 0.86
**Δ Lexical Retrieval**	β = 0.37, SE = 0.7, *p* = 0.60	β = − 0.32, SE = 0.81, *p* = 0.70	β = − 0.44, SE = 0.71, *p* = 0.54
**Δ Fluency**	β = 1.28, SE = 0.72, *p* = 0.08	β = 0.19, SE = 0.84, *p* = 0.82	β = − 0.94, SE = 0.74, *p* = 0.21
**Δ Executive Function**	β = 1.23, SE = 2.97, *p* = 0.68	β = 3.07, SE = 3.44, *p* = 0.38	β = 0.55, SE = 3.02, *p* = 0.86
	**Carotid**		
	**Compliance**	**Diameter**	**Stiffness**
**Episodic Memory**	β = 0.74, SE = 0.73, *p* = 0.31	β = − 0.13, SE = 0.75, *p* = 0.86	β = − 0.28, SE = 0.77, *p* = 0.72
**Visuospatial Memory**	β = − 1.45, SE = 1.16, *p* = 0.22	β = − 0.10, SE = 1.21, *p* = 0.93	β = 1.23, SE = 1.22, *p* = 0.31
**Working Memory**	β = 0.18, SE = 0.41, *p* = 0.66	β = − 0.10, SE = 0.42, *p* = 0.81	β = − 0.27, SE = 0.43, *p* = 0.53
**Lexical Retrieval**	β = 1.63, SE = 0.69, *p* = 0.02	β = − 0.93, SE = 0.73, *p* = 0.20	β = − 1.93, SE = 0.73, *p* = 0.01
**Fluency**	β = 1.53, SE = 0.77, *p* = 0.05	β = 0.01, SE = 0.81, *p* = 1.00	β = − 1.72, SE = 0.8, *p* = 0.03
**Executive Function**	β = − 1.69, SE = 3.28, *p* = 0.61	β = 0.66, SE = 3.38, *p* = 0.85	β = 1.12, SE = 3.44, *p* = 0.75
**Δ Episodic Memory**	β = 0.33, SE = 0.68, *p* = 0.62	β = − 0.46, SE = 0.70, *p* = 0.51	β = 0.38, SE = 0.72, *p* = 0.59
**Δ Visuospatial Memory**	β = − 2.09, SE = 1.19, *p* = 0.09	β = − 2.49, SE = 1.22, *p* = 0.04	β = 1.55, SE = 1.27, *p* = 0.22
**Δ Working Memory**	β = − 0.55, SE = 0.32, *p* = 0.09	β = − 0.28, SE = 0.34, *p* = 0.41	β = 0.37, SE = 0.34, *p* = 0.27
**Δ Lexical Retrieval**	β = 0.86, SE = 0.55, *p* = 0.12	β = − 0.86, SE = 0.57, *p* = 0.12	β = − 0.49, SE = 0.58, *p* = 0.40
**Δ Fluency**	β = 1.38, SE = 0.57, *p* = 0.02	β = − 0.32, SE = 0.61, *p* = 0.60	β = − 1.28, SE = 0.60, *p* = 0.04
**Δ Executive Function**	β = − 2.33, SE = 2.55, *p* = 0.36	β = 0.62, SE = 2.65, *p* = 0.82	β = 1.55, SE = 2.70, *p* = 0.57

Note. Arterial compliance (mm^2^/kPA). Arterial diameter (mm)

All models were adjusted for sex, years of education, age, and height at MRI-Wave-2. Carotid models were also adjusted for ultrasound scanner. Models investigating change in cognitive measures between MRI-Wave-1 and MRI-Wave-2 were also adjusted for time between the two testing sessions. Measures refer to MRI-Wave-2 unless otherwise stated. ß, Unstandardised regression coefficients, SE Standard errors, p, *p* values prior to multiple comparisons correction. Uncorrected significant associations are present in bold. ^*^indicates p_corr_ < 0.05, corrected for multiple comparisons

### Association of aortic measures with cerebrovascular and cognitive outcomes

**Fig. 2:**
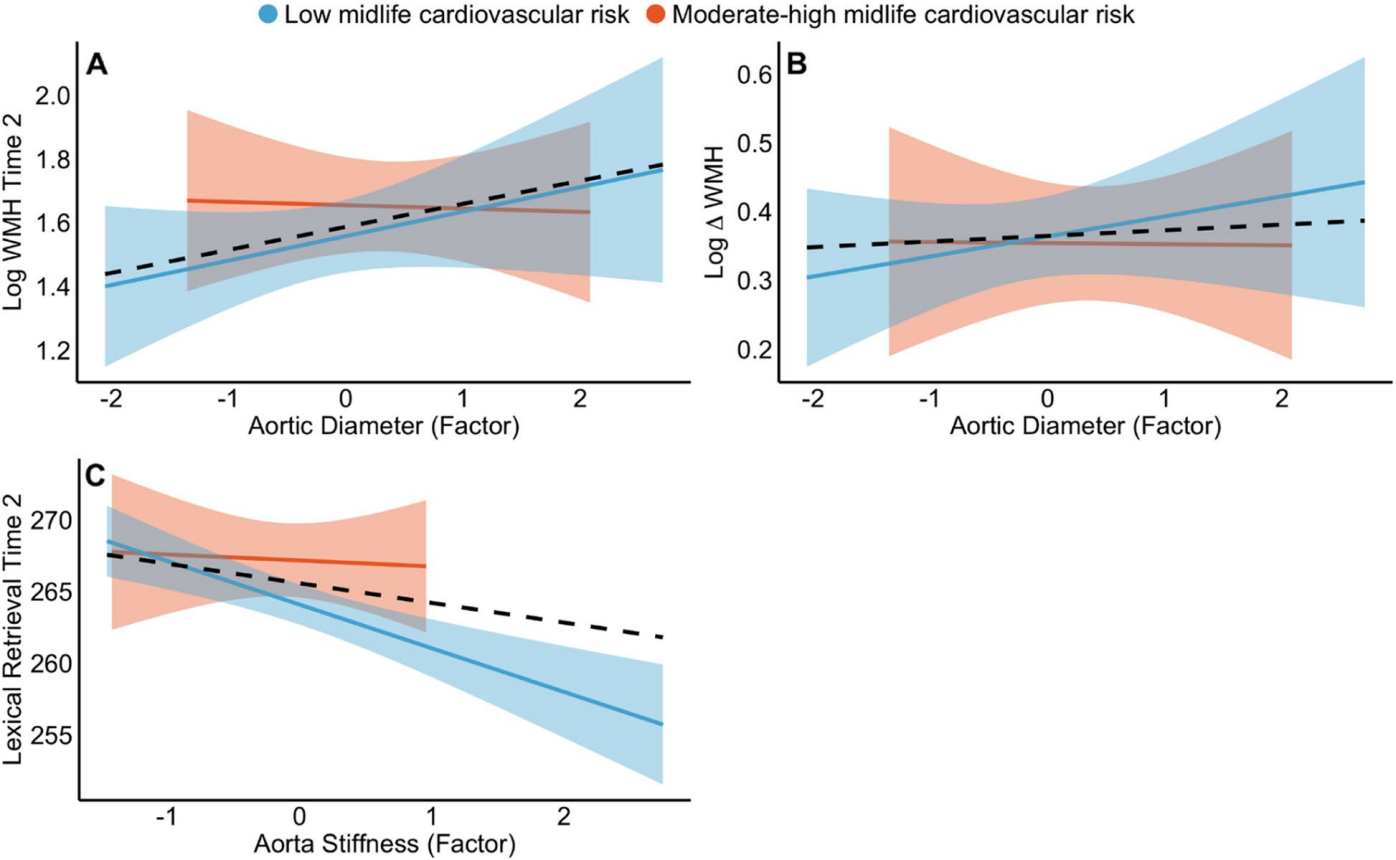
Statistically significant associations between aorta structure with **A** white matter hyperintensities (WMHs) at MRI-Wave 2, **B** change in WMHs, and **C** lexical retrieval at Wave-2. Low and moderate-high midlife FRS are represented in blue and orange, respectively and there were no moderating effects of FRS on these associations. Solid lines represent regression lines with 95% confidence intervals. Black dashed lines represent mean regression lines across cardiovascular risk groups

Larger aortic diameter at MRI-Wave 2 was associated with greater volume of WMHs at MRI-Wave-2 and a greater increase in WMH volume between MRI-Wave-1 and 2 (ß = 0.08, 95% CI [0.02 0.13], *p* = 0.008, *p*_corr_ < 0.05; Table 3 and Fig. 2). There were no significant associations between aorta structure and grey matter CVR at Wave 2.

Aortic stiffness was associated with reduced lexical retrieval at MRI-Wave 2 (ß = − 2.68, 95% CI [− 4.34 − 0.97], *p* = 0.003, *p*_corr_ < 0.05; Table 4 and Fig. 2).

**Fig. 3:**
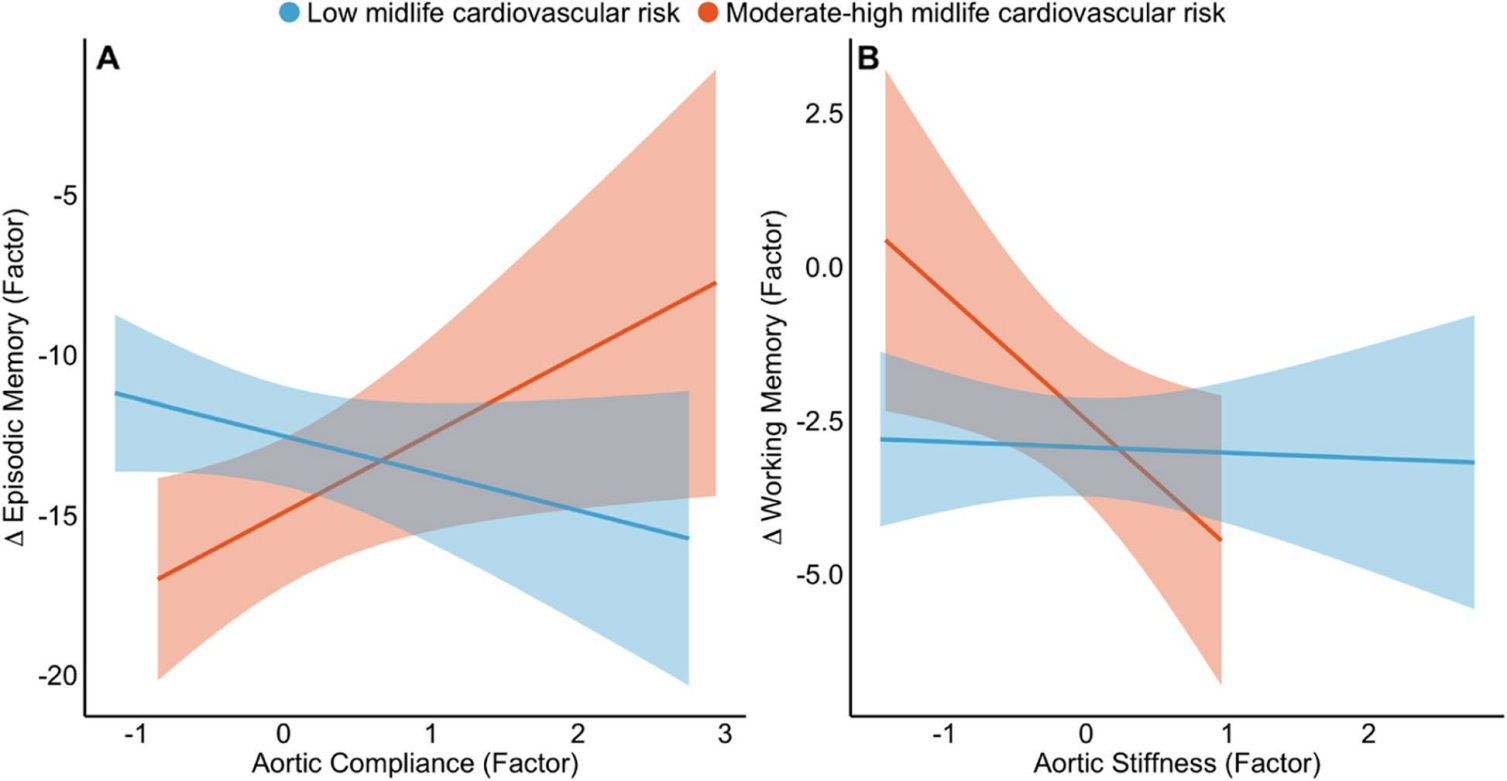
Statistically significant moderating effects of FRS on the association between **A** aortic compliance and change in episodic memory and **B** aortic stiffness and change in working memory. Low and moderate-high midlife FRS are represented in blue and orange, respectively. Solid lines represent regression lines with 95% confidence intervals

We observed significant moderating effects of midlife FRS such that the high-risk group had stronger associations between aorta structure (measured at Wave-2) and longitudinal cognitive decline (from Waves 1 to 2) relative to the low-risk group (Fig. 3). There was a significant interaction between FRS and aortic compliance (ß = 3.28, 95% CI [0.33 6.24], *p* = 0.03), such that higher aortic compliance was linked to less longitudinal decline in episodic memory only in the high-risk group, whereas the low-risk group showed no association between compliance and episodic memory decline (Fig. 3). Similarly, higher aortic stiffness at MRI-Wave-2 was linked to greater longitudinal decline in working memory only in the high-risk group but not the low-risk group (ß = − 2.23, 95% CI [− 4.30 − 0.15], *p* = 0.04; Fig. 3).

### Associations of carotid measures with cerebrovascular and cognitive outcomes

**Fig. 4:**
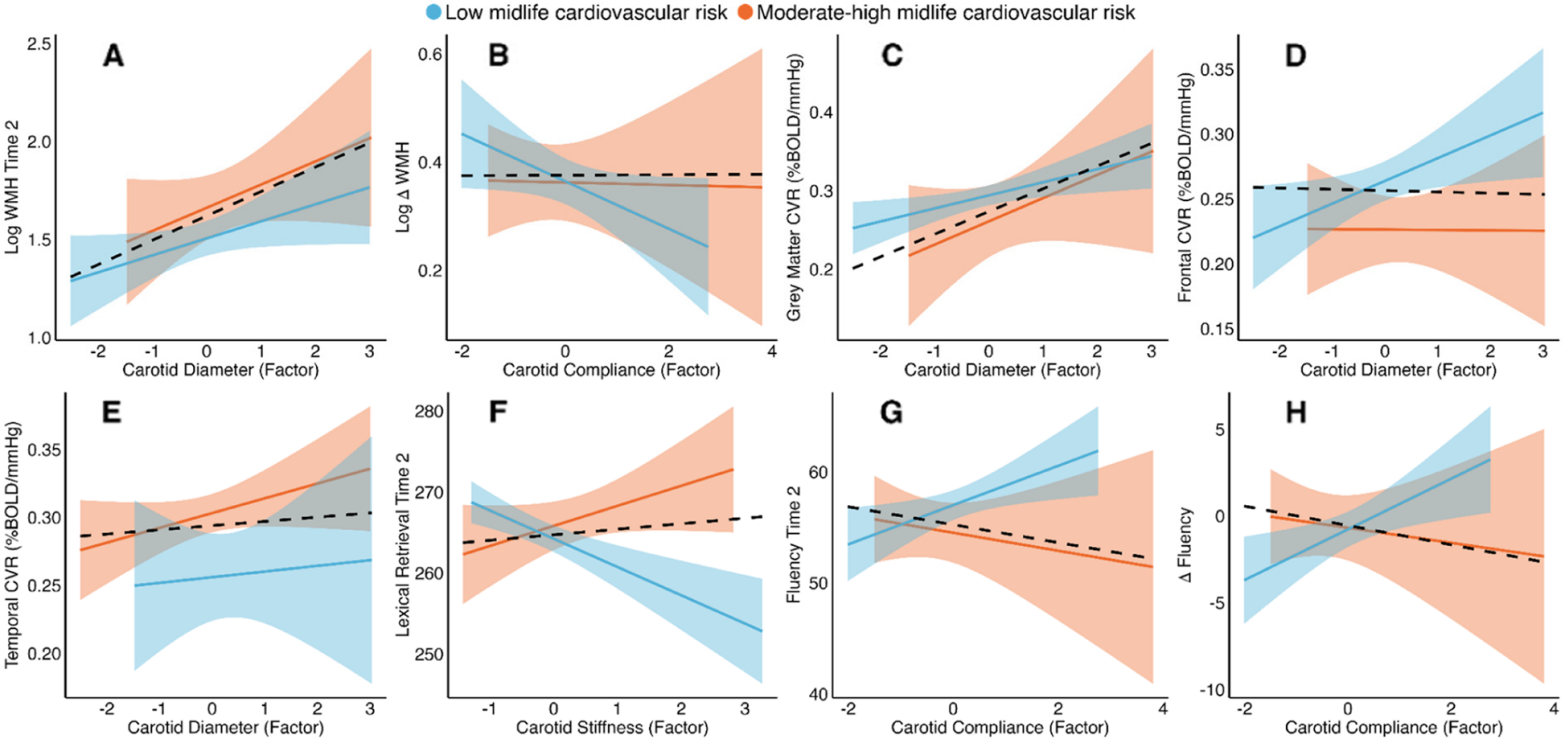
Statistically significant associations between carotid measures and MRI (**A**–**E**) and neuropsychological measures (**F**–**H**). Low and moderate-high midlife FRS are represented in blue and orange, respectively. Solid lines represent regression lines with 95% confidence intervals. Black dashed lines represent mean regression lines across cardiovascular risk groups. For Delta Fluency (**H**): *more negative* values correspond to *greater longitudinal decline*

Larger carotid diameter was associated with higher WMH volume at MRI-Wave 2 (ß = 0.11, 95% CI [0.01 0.21], *p* = 0.03, *p*_corr_ > 0.05; Table 3 and Fig. 4). Lower carotid compliance at MRI-Wave-2 was associated with a greater increase in WMH volume between Waves 1 and 2 (ß = − 0.06, 95% CI [− 0.09 − 0.01], *p* = 0.02, *p*_corr_ < 0.05; Table 3 and Fig. 4).

Larger carotid diameter was associated with higher global grey matter CVR at MRI-Wave-2 (ß = 0.02, 95% CI [0.02 0.03], *p* = 0.04, *p*_corr_ > 0.05; Table 3 and Fig. 4). Post hoc analyses of regional CVR revealed that this effect was present in the frontal lobe (ß = 0.02, 95% CI [0.00 0.04], *p* = 0.02; Table 3 and Fig. 4) and temporal lobe grey matter (ß = 0.02, 95% CI [0.00 0.03], *p* = 0.04; Table 3 and Fig. 4). There was no interaction with the FRS group.

Higher carotid stiffness and lower carotid compliance were associated with lower lexical retrieval and fluency at MRI-Wave 2 as well as greater declines in fluency between MRI-Waves 1 and 2. These associations did not survive corrections for multiple comparisons (summary statistics in Table 4; Fig. 4).

**Fig. 5:**
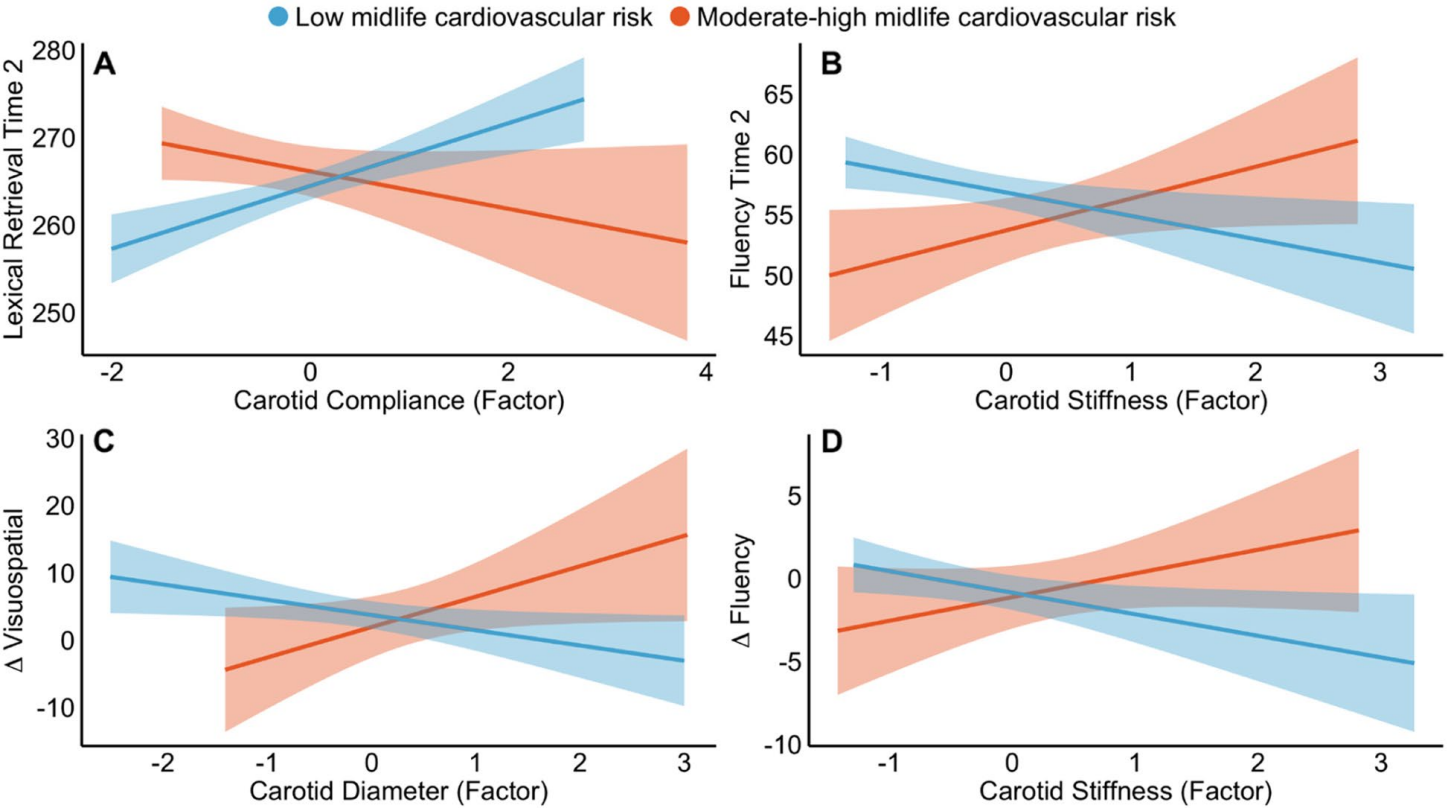
Statistically significant moderating effects of FRS on the association between **A** carotid compliance and lexical retrieval at follow-up, **B** carotid stiffness and fluency at follow-up, **C** carotid diameter and longitudinal change in visuospatial memory, and **D** carotid stiffness and longitudinal change in fluency scores. Low and moderate-high midlife FRS are represented in blue and orange, respectively. For Δ Visuospatial (**C**): *more positive* values correspond to *greater longitudinal decline*, whereas for Δ Fluency (**D**): *more negative* values correspond to *greater longitudinal decline*. Solid lines represent regression lines with 95% confidence intervals

We also observed significant moderating effects of FRS such that some carotid-cognition associations were only significant in the low-risk group. For these associations, while the high-risk group showed the opposite pattern to the low-risk group, its associations were not significant. There was a significant interaction between FRS and carotid compliance (interaction ß = − 2.93, 95% CI [− 5.54 − 0.32], *p* = 0.03), such that lower carotid compliance was linked to lower lexical retrieval at Wave-2 only in the low-risk group (Fig. 5). Similarly, higher carotid stiffness was linked to lower fluency at MRI-Wave-2 only in the low-risk group (interaction ß = 4.36, 95% CI [1.10 7.62], *p* = 0.009, Fig. 5). There was also a significant interaction between FRS and carotid diameter (interaction ß = 7.29, 95% CI [2.41 12.19], *p* = 0.004), such that larger carotid diameter at MRI-Wave-2 was linked to greater longitudinal decline in visuospatial memory in the high-risk group, but less memory decline in the low-risk group between MRI-Wave-1 and 2 (Fig. 5C). Finally, higher carotid stiffness at MRI-Wave-2 was linked to greater longitudinal decline in fluency (between MRI-Wave-1 and 2) only in the low-risk group (interaction ß = 3.09, 95% CI [0.62 5.57], *p* = 0.015, Fig. 5D).

## Discussion

Dementia and cardiovascular diseases are major causes of morbidity and mortality in old age. This study makes several novel observations about the heart-brain association, by relating aorta and carotid artery measures with cerebrovascular and cognitive outcomes. First, we found that wider and stiffer aortas were associated with larger carotid diameter, independent of body size. Second, we demonstrated that higher midlife Framingham cardiovascular risk was associated with larger aortic diameter, larger carotid diameter, and increased carotid stiffness in old age. Third, we observed notable artery-brain associations: larger aortic and carotid diameters were associated with larger WMH lesion volumes. Larger aortic diameter and lower carotid compliance at follow-up was also associated with greater longitudinal increases in WMH volumes over the preceding 9 years. Fourth, higher aortic stiffness was associated with poorer lexical retrieval. Similarly for the carotid artery, greater stiffness and lower compliance were linked to poor lexical retrieval and fluency at Wave-2 as well as greater retrospective declines in fluency between Waves 1 and 2. There were also significant moderating effects of FRS on some artery-cognition associations, such that worse aortic structure at follow-up (lower compliance, higher stiffness) was linked to greater longitudinal declines in episodic and working memory only in the high-risk group. Worse carotid structure at follow-up (lower compliance, higher stiffness) was linked to poor lexical retrieval and greater retrospective longitudinal decline in fluency only in the low-risk group. Larger carotid diameter at follow-up was also associated with greater visuospatial memory decline in the high-risk group, with the opposite pattern in the low-risk group. Fifth, larger carotid diameter was associated with higher cerebrovascular reactivity, which raises the possibility of a compensatory physiological mechanism as described below. In our discussion of the carotid-CVR and carotid-cognition results, we highlight that our findings must be interpreted in light of the fact that although they were significant, they did not survive corrections for multiple comparisons.

Stiffer aortas and higher midlife FRS were associated with wider carotid arteries, albeit with a small effect size. Artery stiffening occurs as a natural part of ageing [50] but is accelerated by midlife vascular risk with similar effect sizes to those seen in this study [51, 52]. Increased pulsatile energy from the aorta, resulting from arterial stiffness, may contribute to a widening of the downstream carotid arteries to lower the mechanical impact of the pulse and, in turn, prevent neurological damage and cognitive impairment. Previous evidence supports such vascular remodelling as a mechanism to maintain constant blood flow, particularly in the presence of cumulative cardiovascular burden (in this case, higher FRS in midlife) and arterial stiffening [53, 54].

A similar compensation mechanism, i.e., cerebrovascular reactivity (CVR), exists within the brain’s microvasculature, where cerebral arteries dilate to regulate the brain’s blood supply to meet metabolic demands [55]. CVR has been positioned as a process that can mitigate the damaging effects of aortic stiffening [56, 57]. For example, in a recent study, aortic stiffness was associated with reductions in cerebral blood flow but preserved CVR suggesting that CVR responses may adjust in order to compensate for arterial damage over time [15]. We therefore propose that progressive stiffening of the aorta can contribute to increases in carotid diameter (vascular remodelling) and compensatory neurovascular mechanisms such as higher CVR, as an attempt to regulate constant blood flow and lessen potential neurological damage in the brain. More specifically, it is possible that a stiffer aorta is less able to buffer pulsatile flow across the cardiac cycle, leading to elevated pulse pressure and subsequent hemodynamic stress on the downstream carotid and cerebral arteries. To accommodate increased pulsatile flow, the carotids may remodel to reduce wall shear stress, so that a larger carotid diameter offers lower vascular resistance and smoother flow of blood to the brain. Similarly, the cerebral arterioles may also dilate to maintain blood flow to the brain in the face of higher pulsatile energy, by enhancing their endothelial sensitivity to vasodilatory stimuli (in this case, CO_2_), resulting in a higher CVR response. That said, it is also possible that increased carotid diameter represents an adaptive response to concomitant carotid stiffening (and alterations in carotid wall shear and tensile stress). Although we lacked the statistical power to test these explanations using mediation analysis, we provide the first evidence showing that aortic stiffening was linked to larger carotid diameter, which in turn was linked to higher CVR, particularly in the frontal and temporal grey matter.

Previous literature also suggests that when vascular remodelling is present but insufficient, high pulsatile pressure transmitted to the cerebral arteries can contribute to microvascular damage in the brain, observable with typically small effect sizes on brain MRI as increased WMH load [58, 59]. Studies have linked aortic measures to WMHs [4, 6, 60] and cognitive decline [14, 61, 62], and here we present additional associations between carotid artery measures and WMH outcomes (with similar effect sizes). We observed a small association between larger aortic and carotid diameter with higher volume of WMHs. Larger aortic diameter and smaller carotid compliance were also associated with a greater longitudinal increase in WMH volume. Compliance is the ability of the vessel wall to distend and regulate the volume of blood flow. Lower carotid compliance, or reduced vessel elasticity, may progressively increase the pressure of blood flowing to the brain, and over time, lead to faster accumulation of WMH lesions.

Loss of elasticity of both the aorta and carotid arteries was also associated with worse cognitive outcomes in the overall sample. Specifically, lower carotid compliance and higher aortic and carotid stiffness were associated with lower cross-sectional lexical retrieval and fluency and greater longitudinal declines in fluency. Interestingly, the higher and lower FRS groups tended to have opposite patterns for all cognitive outcomes. While these moderations may be affected by small sample sizes in the higher FRS group (~ 23% of the sample), potentially contributing to greater variability in this group, it is still worth noting that the links between poor aortic structure (lower compliance, higher stiffness) and greater longitudinal episodic and working memory decline were actually driven by this smaller high-risk group. This suggests that cumulative exposure to cardiovascular risk factors throughout mid-to-old age may indeed exacerbate cognitive decline that stems from arterial stiffening.

Several limitations of this study should be noted. First, the findings may not be widely generalised as the sample is predominantly well-educated, healthier-than-average, male, and Caucasian, reflecting the demographics of the 1985 British Civil Service, from which this cohort was originally drawn. Second, the study’s long duration may introduce survival bias. Third, due to a change in the ultrasound scanner partway through the study, the analyses of the aorta could only be tested in a subset, leading to small sample sizes, especially in aorta analyses. Fourth, caution is needed in interpreting results that did not survive our strict corrections for multiple comparisons, which we have nonetheless reported to facilitate the testing of these hypotheses in future datasets. Fifth, although we present plausible mechanistic explanations for our results, these should be interpreted within the context of our cross-sectional design, which limits us from making any inferences about causation. Since artery metrics were measured at follow-up and the decline in WMH and cognitive performance was assessed retrospectively, we emphasize that artery measurements could not be used as a predictor of cognitive decline, but rather as a correlate of ongoing neurocognitive deterioration. Therefore, while we have suggested a directional pathway, which is consistent with causal models, from aorta to carotid to cerebrovascular and cognitive function when interpreting our results in the context of current evidence, we emphasise that our findings are associative and hence do not lend themselves to any causal directions, nor do they preclude any reverse causation effects.

## Conclusions

In summary, by combining vascular ultrasound, cerebrovascular MRI, and cognitive testing we observed that large artery measures were significantly associated with cerebrovascular measures and cognition. Our findings are consistent with the hypothesis that higher pulsatile energy ascending from the aorta (presenting as higher arterial stiffness) may lead to the widening of upstream vessels (i.e., larger carotid diameter), which in turn may be linked to compensatory regulation of cerebral blood supply (i.e., higher cerebrovascular reactivity). It is likely however that compensatory cascades may not always be sufficient to counter the accumulated exposure to increased pulsatile energy, and loss of elasticity of the aortic and carotid vessel walls may also be associated with higher cerebrovascular lesion load, greater longitudinal increases in WMH lesions, and poorer cognitive performance in older age. Our study adds to evidence supporting the idea that structural integrity of the major arteries could have beneficial effects on brain and cognition in later life.

## Supplementary Information

Additional file 1. Method S1. Ascending aortic artery sonography acquisition. Method S2. Common carotid arteries sonography acquisition. Method S3. Arterial measures calculations. Method S4. Neuroimaging measures calculations. Method S5. Details of cognitive measures. Figure S1. Flowchart showing participant inclusion in each subset of analyses. Figure S2. Principal component analyses of cognitive, aortic, and carotid measures. Figure S3. Correlationsbetween carotid and aortic measures acquired at MRI-Wave-2. Table S1. Demographic characteristics and exposures between risk groups. Table S2. Descriptive statistics on outcome data for overall sample between MRI-Wave-1 and MRI-Wave-2. Table S3. Supplementary analyses reproduced in a consistent sample size across all outcome variables

## Data Availability

Data from MRI-Wave-1are shared on Dementias Platform UK (DPUK) portal (https://portal.dementiasplatform.uk/Apply). As restrictions apply to the availability of these data, which were used under licence for the current study, the authors cannot publicly share this data. However, the data are freely available by application to DPUK. As per study policy, data from MRI-Wave-2 (Heart and Brain Study) will also be made freely available on the DPUK portal within five years after study completion, by 2028.

## Abbreviations

BOLD: Blood-oxygen-level-dependent
CO_2_: Carbon dioxide
CVD: Cardiovascular disease
CVR: Cerebrovascular reactivity
EtCO_2_: End-tidal CO_2_
FRS: Framingham Cardiovascular Risk Score
FSL: FMRIB Software Library
MRI: Magnetic resonance imaging
PCA: Principal component analysis
WMHs: White matter hyperintensities
