# Supplementary material for: Associations of aortic and carotid artery health with cerebrovascular markers and cognition in older adults from the Whitehall II imaging study
Source: BMC Med. 2025 Jun 3;23:330. doi: 10.1186/s12916-025-04105-y (PMC12135225; doi:10.1186/s12916-025-04105-y)
Supplement: Supplementary file 1 — Additional file 1. Method S1. Ascending aortic artery sonography acquisition. Method S2. Common carotid arteries sonography acquisition. Method S3. Arterial measures calculations. Method S4. Neuroimaging measures calculations. Method S5. Details of cognitive measures. Figure S1. Flowchart showing participant inclusion in each subset of analyses. Figure S2. Principal component analyses of cognitive, aortic, and carotid measures. Figure S3. Correlationsbetween carotid and aortic measures acquired at MRI-Wave-2. Table S1. Demographic characteristics and exposures between risk groups. Table S2. Descriptive statistics on outcome data for overall sample between MRI-Wave-1 and MRI-Wave-2. Table S3. Supplementary analyses reproduced in a consistent sample size across all outcome variables [file 12916_2025_4105_MOESM1_ESM.docx]

**Additional detail on vascular sonography acquisition.**

**Method S1. Ascending aortic artery.**

Participants were asked to rest in the left lateral decubitus position for data acquisition. Measurements were taken using a 7S array transducer (frequency 6.0 MHz). We acquired 20-30 heartbeats of continuous M-Mode data in the parasternal long axis view. Blood velocity was measured over a similar timeframe in the apical five-chamber view using pulse-wave Doppler.

**Method S2. Common carotid arteries.**

Participants were asked to rest in the supine position for data acquisition. Measurements were taken using an M12L linear array transducer (centre frequency 14.0 MHz). We acquired longitudinal B-mode images in the ear-to-ear plane at a location 1-2 cm proximal to the carotid bifurcation. We measured carotid wall motion for between 20-30 heart beats for each participant on both the left and right sides. We acquired duplex Doppler images from the same location for an addition 20-30 heart beats. Beam insonation angle was maintained at <60 degrees to allow for estimation of the blood velocity waveform. Volumetric blood flow as estimated using the measured diameter.

**Method S3. Arterial measures**

The following measures were calculated for both the aorta and the left and right internal carotid arteries using the formulae provided. Measures were acquired separately for the left and right internal carotid arteries and then averaged to generate a single metric for the carotid arteries overall.

- **Diameter:** Diameter of the artery, measured from the inner arterial walls.
  - Calculation: Mean of five measures.
- **Pulse wave velocity:** Local arterial stiffness, or the rate at which pressure waves move along the artery.
  - Calculation: Bramwell Hill Equation = SQRT(1/Rho*Distensibility)
  - Note that Rho represents the standard density of blood (i.e., 1060 kg/m^3^)
- **Compliance:** Change in arterial diameter, relative to the amount of pressure exerted on the arterial walls.
  - Calculation: [Pi * (D_sys_^2^ – D_dia_^2^)]/4*Pulse Pressure
- **Distensibility:** The extent to which the artery expands from its resting value, relative to the pressure applied.
  - Calculation: 2 x Strain / Pulse Pressure
    - Strain = (D_sys_ – D_dia_)/D_dia_
- **Beta stiffness:** Local arterial stiffness, independent of blood pressure.
  - Calculation: [Ln(SBP/DBP)]/Strain

**Method S4. Neuroimaging measures**

The following measures were calculated to characterise brain health. A brief description of each measure is provided below.

- **Global CVR:** The percentage change in BOLD signal per mmHg change in EtCO_2_ (%BOLD /1EtCO_2_ mmHg) across the grey matter.
- **Regional CVR:** The percentage change in BOLD signal per mmHg change in EtCO_2_ (%BOLD /1EtCO_2_ mmHg) measured separately for the frontal, parietal, occipital and temporal lobes. This was used in post hoc analyses only if global CVR was significantly associated with the variable of interest in order to establish whether changes in any specific lobe were driving the global change.
- **WMH volume:** The normalised volume of WMHs across the brain, expressed as a percentage of the total brain volume. Values were log transformed due to the skewed distribution of data.
- **Change in WMH volume:** The change in the log-transformed and normalised volume of WMHs between MRI-Wave-1 and 2 (i.e., WMH volume at MRI-Wave-2 – WMH volume at MRI-Wave-1).

**Method S5. Cognitive measures**

The following cognitive tasks were administered during MRI-Wave-1 and 2. A brief description of each task is provided, alongside the specific measure from the task that was used in statistical analyses.

- **Trail Making Test Versions A and B**: In Version A, participants were asked to draw a line to connect letters in order as quickly and accurately as possible. In Version B, participants were asked to draw a line to connect both letters and numbers in order, alternating between letters and numbers, as quickly and accurately as possible.
- **Hopkins Verbal Learning Test-Revised**: Participants were read a list of twelve nouns three times and asked to recall as many words as possible immediately after each presentation (HVLT TR), after a longer delay period (HVLT DR), and from force choice alternatives after a delay period (HVLT RDI).
- **Digit Span Test**: This task was taken from Wechsler’s Adult Intelligence Scale Version IV. Across three tasks, participants were read a series of strings of numbers that increase in length and asked to (1) recall the numbers exactly as they were read (DSF), (2) recall the numbers in backwards order (DSB), and (3) recall the numbers in ascending numerical order (DSS).
- **Digit Coding Test:** This task was taken from Wechsler’s Adult Intelligence Scale Version IV. Participants were given a key showing marks corresponding to each number (1-9). Participants were presented with a grid containing numbers and asked to work their way through the grid, filling in the marks corresponding to the numbers, as quickly and accurately as possible. They were given 120 seconds to fill in as many marks as possible (DCOD).
- **Category Fluency Test:** This task was taken from Addenbooke’s Cognitive Examination Revised. Participants were given 60 seconds to say as many words as they could beginning with the letter ‘F’ (CF L). Participants were then given 60 seconds to say as many animals as they could (CF C).
- **Rey Complex Figure Test and Recognition Trial**: Participants were shown a complex line drawing and asked to copy it as accurately as possible (RCF COP). After copying the drawing, it was removed, and participants were asked to recreate it from memory and scored on accuracy (RCF IMM). Participants were scored again when asked to recreate the drawing from memory again after a longer delay (RCF DEL).
- **Boston Naming Test**: Participants were shown a series of line drawings depicting objects and scored on number of correctly named objects (BNT).
- **Test of Premorbid Functioning:** Participants were shown a list of words and scored on Total number of words read aloud correctly scaled to full scale IQ (TOPF).

Based on the scree plots and eigenvalues we obtained six cognitive factors representing episodic memory (HVLT-TR, HVLT-DR, and HVLT-RDI), visuospatial memory (RCF-COP, RCF-IMM, RCF-DEL), executive function (positive loadings of TMTA and TMTB and negative loadings of DCOD), working memory (DSF, DSB, DSS), fluency (FL-C and FL-L), and lexical retrieval (BNT and TOPF). Better performance is reflected as higher values for the episodic memory, fluency, and lexical retrieval domains, and lower values on the visuospatial and executive function domains.

**Figure S1.** Flowchart showing participant inclusion in each subset of analyses. Participants (*n*=163) were recruited to the Heart and Brain Study between November 2019-January 2023, with a break in testing due to the COVID-19 pandemic (March 2020-Janurary 2022). Carotid data (*n*=153) were collected from November 2019-January 2023. Aortic data (*n*=89) were collected between November 2019-January 2023, with a change in scanner preventing aortic data being collected for participants recruited between January 2023-May 2023. Given the constraints in sample size, we used the best available data for each analysis presented in the main text (shown in boxes with orange solid lines). However, we also performed a supplementary analysis using a consistent sample size across all outcomes (shown in boxes with blue dotted lines). The results of the supplementary analyses are presented in **Table S3** and are consistent with the main analyses.

**
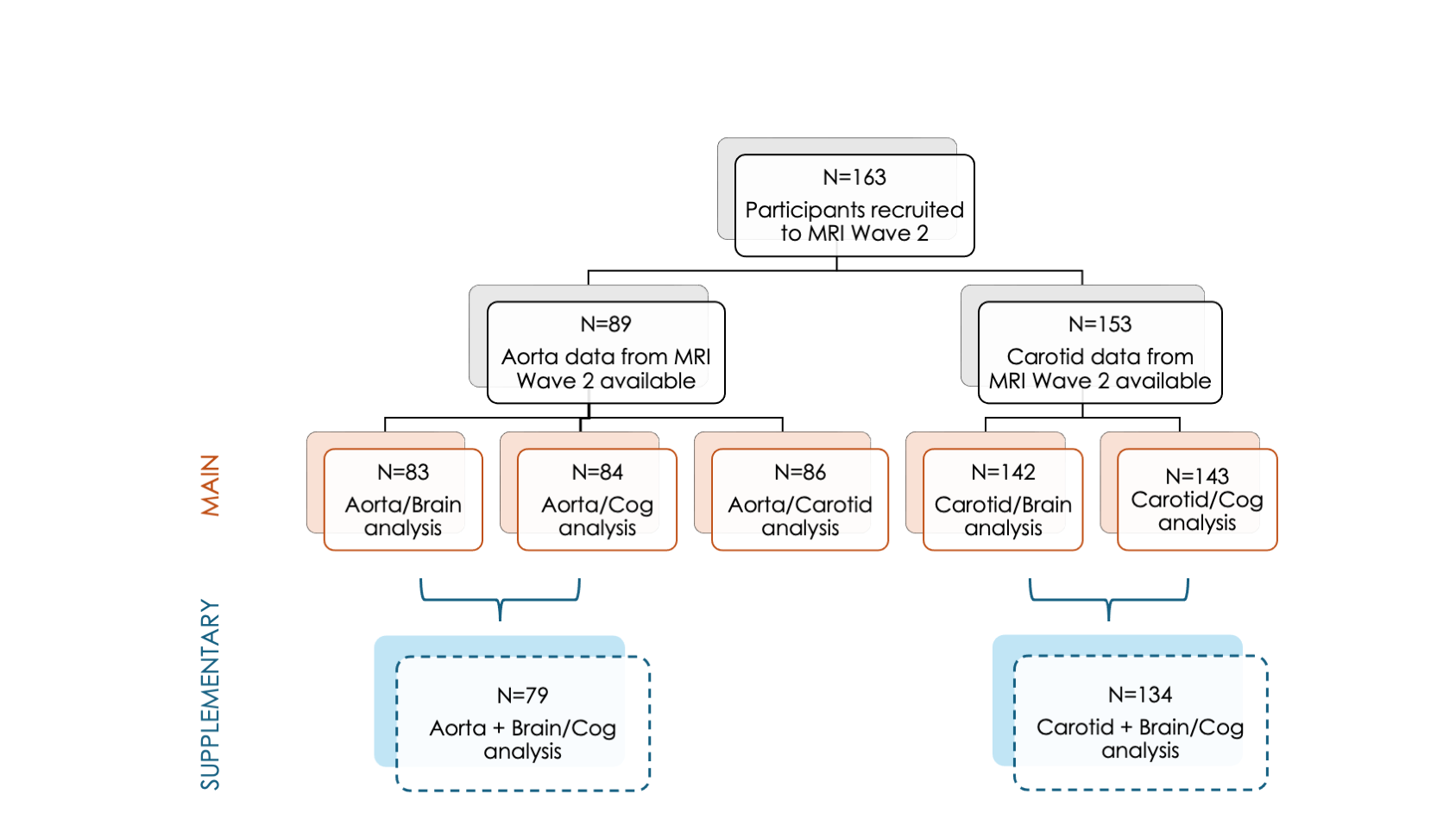
**

**Figure S2.** Principal component analyses (PCAs) conducted to summarise cognitive, aortic, and carotid measures. Presented are (A) Scree plots showing the eigenvalues for PCA components identified, (B) Factor loadings for aortic measures, (C) Factors loadings for carotid measures, and (D) Factors loading for cognitive measures. Acronyms used to describe cognitive measures are described in the Method S5 above.


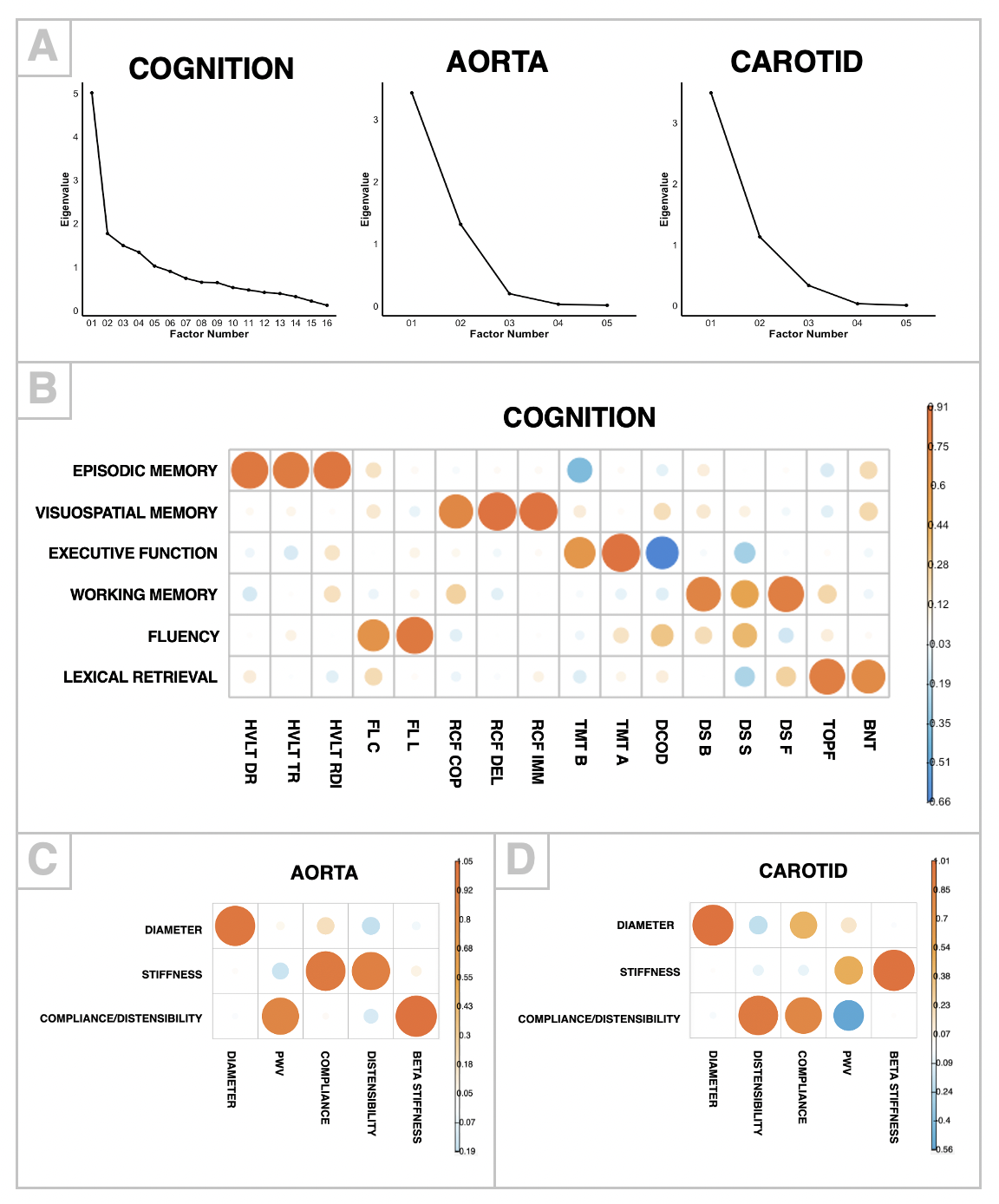


**Figure S3.** Correlations (r values) between carotid and aortic measures acquired at MRI-Wave-2 (red=positive correlations, blue = negative correlations).

**
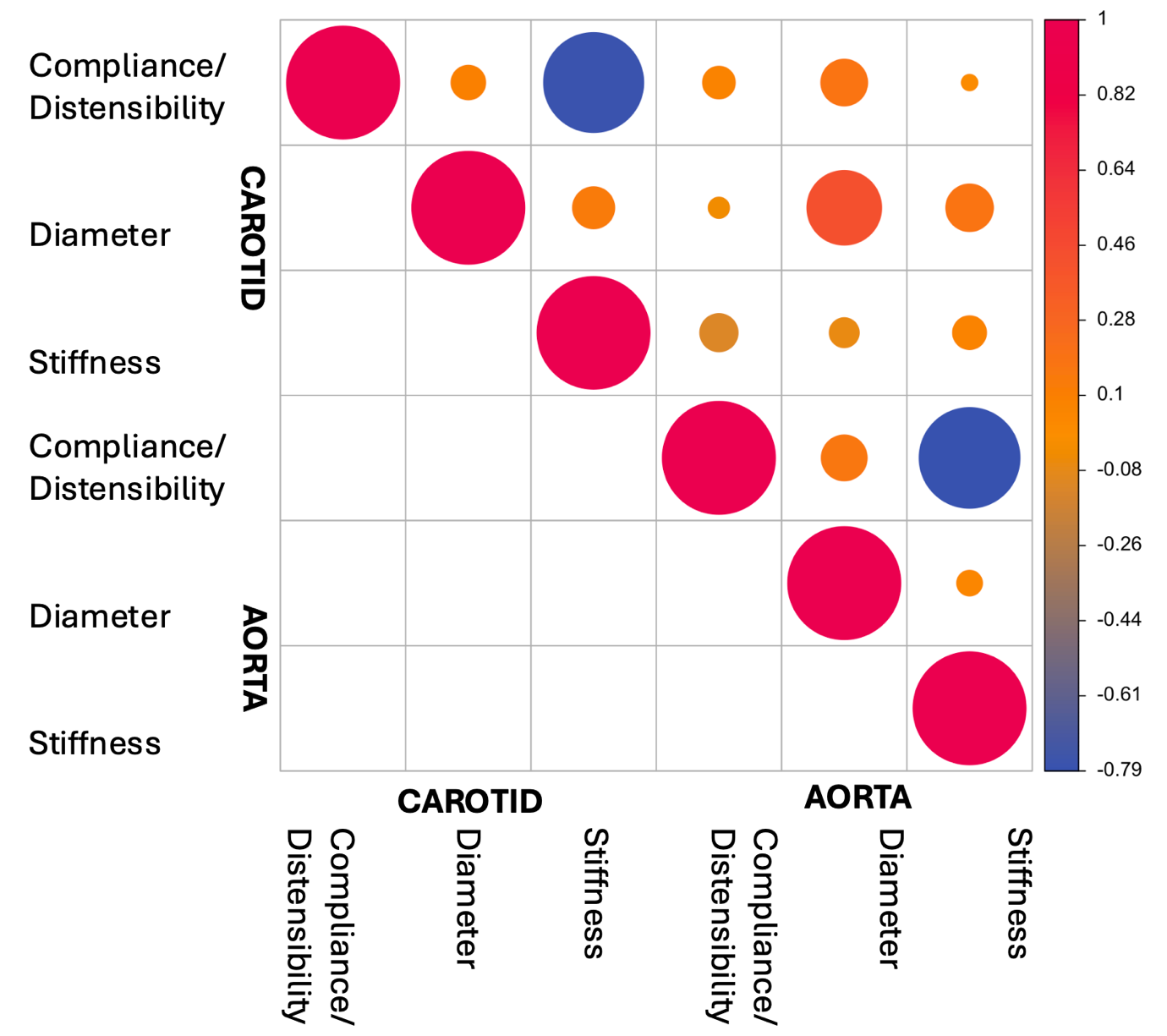
**

**Table S1.** Demographic characteristics and exposures for the overall sample (n=163), stratified by low Framingham scores (FRS) in midlife (*n*=124) and moderate-high Framingham scores (FRS) in midlife (*n*=38), with n=1 participant excluded due to missing FRS values. Values were obtained at MRI-Wave-2 unless otherwise specified.

| **Variable** | **Low FRS**  **N = 124** | **Moderate-High FRS**  **N = 38** |
| --- | --- | --- |
| Gender, *N* (%) |  |  |
| *Female* | 40 (32.3) | 1 (2.6) |
| *Male* | 84 (67.7) | 37 (97.4) |
| Age at MRI-Wave-2 (years), *M* (*SD*) | 75.8 (3.9) | 80 (4.6) |
| Education (years), *M* (*SD*) | 15 (3.5) | 15.1 (3.7) |
| Time between MRI-Wave-1 and MRI-Wave-2 testing (years), *M* (*SD*) | 8.7 (1.2) | 8.5 (1.4) |
| BMI at MRI-Wave-2 (kg/m^2^), *M* (*SD*) | 25.6 (4) | 26.8 (5) |
| Systolic BP (mm/Hg), *M* (*SD*) | 149.3 (19.9) | 150.8 (17) |
| Diastolic BP (mm/Hg), *M* (*SD*) | 78.8 (11.3) | 77.3 (9) |
| Pulse pressure, *M* (*SD*) | 70.5 (16) | 73.5 (15.9) |
| Ultrasound scanner, *N* (%) |  |  |
| *GE VIVID 7* | 76 (61.3) | 35 (92.1) |
| *ZONARE Z.One* | 48 (38.7) | 3 (7.9) |
| Aortic diameter (mm), *M* (*SD*) | 34.1 (3.6) | 36.3 (3.7) |
| Aortic pulse wave velocity (ms), *M* (*SD*) | 12.9 (3.7) | 12.6 (3.3) |
| Aortic compliance (mm^2^/kPA), *M* (*SD*) | 6.3 (3.8) | 6.5 (4) |
| Aortic distensibility coefficient, *M* (*SD*) | 7.2 (3.9) | 7 (4.2) |
| Aortic beta stiffness index, *M* (*SD*) | 25.7 (15) | 24.4 (12.4) |
| Carotid diameter (mm), *M* (*SD*) | 79.1 (8.5) | 83.1 (8.4) |
| Carotid pulse wave velocity (ms), *M* (*SD*) | 8.2 (3.8) | 10.2 (3.8) |
| Carotid compliance (mm^2^/kPA), *M* (*SD*) | 0.6 (0.2) | 0.5 (0.2) |
| Carotid distensibility coefficient, *M* (*SD*) | 11.8 (3.4) | 10.1 (3.7) |
| Carotid beta stiffness index, *M* (*SD*) | 13.1 (3.8) | 15.6 (4.7) |

**Table S2.** Descriptive statistics summarising outcome data for the overall participant sample (n = 163) from MRI-Wave-1 and MRI-Wave-2. Data on sample demographics and exposures are presented in the main manuscript in Table 1.

|  | **MRI-Wave-I** | **MRI-Wave-2** |
| --- | --- | --- |
| **Neuroimaging measures** |  |  |
| Log WMH volume | 1.20 (0.40) | 1.55 (0.49) |
| Global grey matter CVR (%BOLD/mmHg) | NA | 0.29 (0.08) |
| **Cognitive measures** |  |  |
| HVLT-R Delayed Recall | 9.96 (2.28) | 8.10 (2.99) |
| HVLT-R Immediate Recall | 29.07 (3.83) | 23.58 (5.038) |
| HVLT-R Recognition Discrimination | 11.05 (1.2) | 9.87 (1.9) |
| Fluency (Category - Animals) | 23.17 (5.35) | 22.67 (7.00) |
| Fluency (Letter - F) | 16.13 (4.22) | 15.96 (5.01) |
| Rey Complex Figure Test Copy | 31.49 (3.25) | 32.61 (3.80) |
| Rey Complex Figure Test Immediate Recall | 16.88 (5.93) | 16.77 (6.86) |
| Rey Complex Figure Test Delayed Recall | 16.25 (5.80) | 16.66 (6.64) |
| Trail Making Test Version A (seconds) | 28.07 (8.72) | 31.80 (11.37) |
| Trail Making Test Version B (seconds) | 61.39 (25.71) | 76.84 (45.50) |
| Digit Coding Test | 64.88 (12.01) | 60.35 (13.16) |
| Digit Span Test Forwards | 11.12 (2.30) | 11.03 (2.31) |
| Digit Span Test Backwards | 10.20 (2.60) | 9.44 (2.54) |
| Digit Span Test Sequencing | 10.55 (2.18) | 8.84 (2.25) |
| Digit Span Test Total | 31.87 (5.63) | 29.31 (5.47) |
| Boston Naming Test | 57.74 (4.22) | 56.98 (4.37) |
| Test of Premorbid Functioning Scaled Score | 118.77 (8.76) | 116.019 (9.52) |

**Table S3:** Supplementary analysis to determine reproducibility of results in a consistent sample size of n=79 for the Aorta analysis and n=134 for the Carotid analysis. The significant results (p<0.05) from the main text are reanalysed here. The results that were not significant (p>0.05) in the main text had very similar summary statistics in the supplementary analysis and hence are not presented again here. WMH = white matter hyperintensities, CVR = cerebrovascular reactivity, FRS = Framingham Risk Score.

| **Main Effects** | | |
| --- | --- | --- |
| **Dependent variable** | **Independent variable** | **Summary statistics** |
| WMH | Aorta Diameter | β = 0.14, *SE* = 0.06, *p =* 0.02 |
| Δ WMH | Aorta Diameter | β = 0.07, *SE* = 0.03, *p =* 0.03 |
| Lexical Retrieval | Aorta Stiffness | β = -2.37, *SE* = 0.80, *p =* 0.004 |
| WMH | Carotid Diameter | β = 0.08, *SE* = 0.05, *p =* 0.09 |
| Δ WMH | Carotid Compliance | β = -0.06, *SE* = 0.02, *p =* 0.01 |
| Whole-brain CVR | Carotid Diameter | β = 0.02, *SE* = 0.01, *p =* 0.05 |
| Frontal Lobe CVR | Carotid Diameter | β = 0.02, *SE* = 0.01, *p =* 0.02 |
| Temporal Lobe CVR | Carotid Diameter | β = 0.02, *SE* = 0.01, *p =* 0.06 |
| Lexical Retrieval | Carotid Compliance | β = 1.88, *SE* = 0.67, *p =* 0.006 |
| Lexical Retrieval | Carotid Stiffness | β = -2.05, *SE* = 0.70, *p =* 0.004 |
| Fluency | Carotid Compliance | β = 1.76, *SE* = 0.78, *p =* 0.03 |
| Fluency | Carotid Stiffness | β = -1.88, *SE* = 0.80, *p =* 0.02 |
| Δ Fluency | Carotid Compliance | β = 1.40, *SE* = 0.60, *p =* 0.02 |
| Δ Fluency | Carotid Stiffness | β = -1.27, *SE* = 0.62, *p =* 0.04 |
| Δ Visuospatial | Carotid Diameter | β = -2.43, *SE* = 1.25, *p =* 0.05 |
| **Interaction Effects** | | |
| **Dependent variable** | **Interaction** | **Summary statistics** |
| Δ Working Memory | Aorta Stiffness x FRS | β = -2.35, *SE* = 1.65, *p =* 0.04 |
| Δ Episodic Memory | Aorta Compliance x FRS | β = 3.02, *SE* = 1.81, *p =* 0.10 |
| Lexical Retrieval | Carotid Compliance x FRS | β = -3.40, *SE* = 1.27, *p =* 0.009 |
| Fluency | Carotid Stiffness x FRS | β = 5.02, *SE* = 1.70, *p =* 0.004 |
| Δ Fluency | Carotid Stiffness x FRS | β = 3.13, *SE* = 1.33, *p =* 0.02 |
| Δ Visuospatial | Carotid Diameter x FRS | β = 6.95, *SE* = 2.54, *p =* 0.007 |
